# Postoperative pulmonary complications following posterior spinal instrumentation and fusion for congenital scoliosis

**DOI:** 10.1371/journal.pone.0207657

**Published:** 2018-11-16

**Authors:** Si Yin, Huiren Tao, Heng Du, Chaoshuai Feng, Yimin Yang, Weizhou Yang, Chunguang Duan

**Affiliations:** 1 Department of Orthopaedic Surgery, First Affiliated Hospital of Xi’an Jiaotong University, Xi’an, Shaanxi Province, China; 2 Department of Orthopaedic Surgery, General Hospital of Shenzhen University, Shenzhen, Guangdong province, China; 3 College of Medicine, Xi'an Jiaotong University, Xi'an, Shaanxi Province, China; University of California San Francisco, UNITED STATES

## Abstract

**Background:**

Congenital scoliosis (CS) may lead to more serious pulmonary complications compared with idiopathic scoliosis after spinal fusion surgery. However, little has been reported about postoperative pulmonary complication events in patients with CS after spinal fusion surgery.

**Objective:**

To investigate the incidence of and predictive factors of postoperative pulmonary complications following posterior spinal instrumentation and fusion surgery for the treatment of CS.

**Methods:**

We retrospectively reviewed the records of 174 patients with CS (128 females and 46 males, mean age 16.4 years) treated with posterior spinal instrumentation and fusion surgery between January 2012 and April 2017. We extracted demographic, medical history, and clinical data, and investigated the major predictive factors for postoperative pulmonary complications by logistic regression and receiver-operating characteristic curves analyses.

**Results:**

A total of 26 (14.9%) patients developed postoperative pulmonary complications, consisting of pleural effusion (10.9%), pneumonia (6.9%), pneumothorax (1.1%), atelectasis (2.3%), hypoxemia (6.3%), and respiratory failure (1.1%). Logistic regression analysis revealed that the predictive factors for postoperative pulmonary complications were age > 18.1 years (*P* = 0.039), a Cobb angle of > 77° (*P* = 0.011), operation time of > 430 min (*P* = 0.032), and blood transfusion volume > 1500 ml (*P* = 0.015).

**Conclusions:**

Postoperative pulmonary complications are among the main complications following posterior spinal instrumentation and fusion surgery in patients with CS. Such patients aged >18.1 years, with Cobb angles > 77°, operation times > 430 min, and/or blood transfusion volume of > 1500 ml may be more likely to develop postoperative pulmonary complications.

## Introduction

Congenital scoliosis (CS) is a failure of vertebral formation and/or segmentation arising from abnormal vertebral development during gestation, with an overall incidence of approximately 1 in 1000 live births [1, 2]. In recent decades, remarkable progress has been made in the treatment of scoliosis, but some patients still develop postoperative pulmonary complications, with a reported incidence during hospitalization ranged from 2.3% to 50% [3–6]. In addition to severe and complicated spinal deformity and restrictive lung deficit due to multiple anomalies, CS may lead to more important pulmonary complications including respiratory failure requiring prolonged ventilator support after spinal fusion surgery [7]. Little has been reported about the postoperative pulmonary complication events during initial hospitalization in patients with CS. This retrospective study was undertaken to investigate the prevalence of and predictive factors related to postoperative pulmonary complications following posterior spinal instrumentation and fusion surgery for the treatment of CS.

## Materials and methods

### Patients

We reviewed the hospital and clinical records of 174 patients with CS (46 males, 128 females; mean age 16.4 years, range 4.8–44.6 years) who underwent posterior spinal instrumentation and fusion surgery between January 2012 and April 2017. The Ethics Sub-Committee of First Affiliated Hospital of Xi’an Jiaotong University approved this research. Written consents were obtained from all the subjects, and when applicable from their guardians.

CS was classified as resulting from the failure of formation, failure of segmentation, or mixed abnormality (mixed and complex lesions) [1]. Information collected from patients’ records included the admission number, age, weight, sex, indications for surgery, preoperative medical conditions, perioperative details, anesthesia records, perioperative complications, and laboratory findings.

### Clinical and radiological assessment

Before surgery, patients’ preoperative conditions were assessed by a series of examinations including a complete blood cell count, measurement of the erythrocyte sedimentation rate and C-reactive protein level, pulmonary function testing (PFT), and abdominal ultrasound and echocardiography. PFT measured five parameters: forced vital capacity (FVC), the FVC ratio, forced expiratory volume (FEV), and FEV at the end of the first second (FEV_1_) and its ratio.

Preoperative radiographs of the patients, including chest X-ray, standing anteroposterior and lateral radiographs of the entire spine, whole-spine magnetic resonance (MR) images, three-dimensional computed tomography (CT) reconstructions, and supine right and left bending radiographs, were also examined. All patients underwent three-dimensional CT and neural-axis MR imaging from the brainstem to the sacrum to detect associated intraspinal anomalies. Cobb angles and coronal and sagittal alignments were measured on long cassette films using C7 as the plumb line reference. Postoperative radiographic data included the degree of spinal curvature correction immediately postoperatively and at the final follow-up.

### Surgical techniques

The same surgeon (H Tao) performed all surgeries. Halo-gravity traction was performed preoperatively in patients with Cobb angles > 100°. Respiratory function training was carried out preoperatively for all surgical patients. All surgeries were performed under general anaesthesia in the prone position. The determined spine levels were exposed through a standard posterior midline approach, and the posterior elements were exposed by subperiosteal dissection extending laterally to the transverse processes. Then, instrumentation and correction of spinal curvature using a posterior fusion technique were performed. The overlying musculature, fascia, and skin were closed in anatomical layers. Occasionally, osteotomies such as Smith-Petersen osteotomy (SPO), pedicle subtraction osteotomy (PSO), and vertebral column resection (VCR) were performed to obtain a balanced spine. The selection of osteotomy methods depended on the curve type, rigidity, and severity of spinal deformity. Osteotomies were generally classified into six resection grades based on Schwab et al.’s [8] system. After thoracic vertebral osteotomy, an intraoperative lung recruitment maneuver was routinely conducted to confirm that no intraoperative pleural injury was detected.

All patients were closely monitored intraoperatively according to transcranial electric motor-evoked potential and somatosensory-evoked potential. After surgery, all patients were engaged in a supervised physical therapy program and given a home exercise protocol.

### Pulmonary complications

Postoperative pulmonary complications were defined as pulmonary abnormalities occurring in the postoperative period, including atelectasis, pleural effusion, pneumothorax, pneumonia, hypoxemia (oxygen saturation (SO_2_) < 90% over 8 h), respiratory failure, and increased requirement for postoperative mechanical ventilation. The presence of any perioperative cardiopulmonary symptoms or signs (dyspnea, breathlessness on exertion, crackles, or rhonchi) were recorded. Chest radiographs obtained within 3 days postoperatively were examined to note the presence of pulmonary complications. Postoperative chest radiographs and thoracic ultrasound images were obtained in patients with abnormal cardiopulmonary symptoms and signs or suspicious chest auscultation findings, when necessary.

### Statistical analysis

Continuous variables are reported as mean and standard deviation. Categorical variables are reported as number and percentages. Univariate analysis with the t test was performed to examine potential risk factors among continuous variables, such as age, weight, PFT variables, blood cell count variables, Cobb angle, fusion levels, operation time, duration of anesthesia, estimated blood loss, volume of blood transfusion and intraoperative infusion, crystalloid-colloid ratio, intraoperative fraction of oxygen inspiration (FiO_2_), and intraoperative tidal volume. Univariate analysis by the chi-square test and Fisher’s exact test was performed to examine potential risk factors among categorical variables, such as sex, grade of osteotomy, and preoperative chest X-ray findings. A multiple logistic regression model was used to identify significant predictors of postoperative pulmonary complications. We generated a receiver-operating characteristic (ROC) curve using predicted probability values from the logistic regression model. The ROC curve was used to evaluate the optimal cutoff value, which was calculated based on the maximal sum of sensitivity and specificity with the bootstrap normal approximation method. Statistical significance was accepted for *P* values of <0.05. All analyses were performed with the use of SAS software (version 9.4; SAS Institute, Cary NC, USA).

## Results

No patient was lost to follow-up. The mean follow-up duration was 27 months (range 6–69 months). The mean operation time was 348.2 min (range 145–660 min), with an average estimated blood loss of 1077.0 ml (range 300–3500 ml). The average number of levels fused during operation was 11.1 (range 5–15). The average preoperative coronal Cobb angle was 69.6° (range 45–144°), which was corrected to 29.1° (range 18–52°) immediately postoperatively, with a correction rate of 55.1%. No obvious loss of correction was observed at final follow-up examinations.

**Fig 1:**
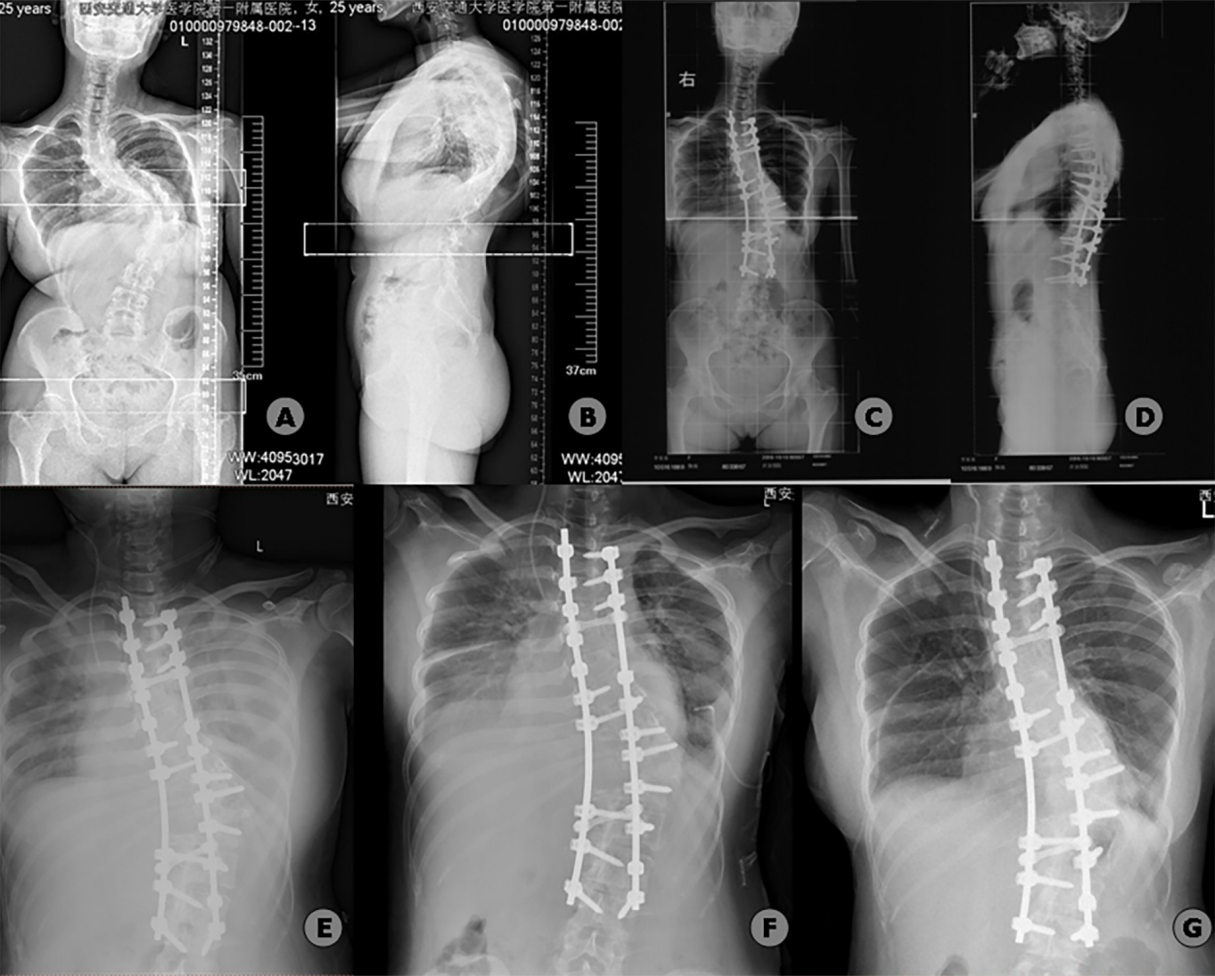
Radiographic findings for a 25-year-old woman with congenital scoliosis who developed postoperative pulmonary complications (bilateral pneumonia and bilateral pleural effusion) after posterior instrumentation, posterior T11 vertebral column resection, and correction and fusion surgery. Preoperative radiographs (A, B) demonstrate a left main thoracic curve of 120°, which was corrected to 38° postoperatively (C, D). The operation time was 450 min, and the volume of intraoperative blood transfusion was 3200 ml. A bedside chest radiograph taken on the third postoperative day (E) and the thoracic ultrasound showed bilateral pneumonia and pleural effusion. After 5 days of treatment with closed thoracic drainage and antibiotics, the patient’s lung condition improved markedly (F). The pulmonary complications had basically recovered by 8 days postoperatively (G).

The incidence of postoperative pulmonary complications is shown in Table 1. Twenty-six (14.9%) of the 174 patients, developed postoperative pulmonary complications, consisting of pleural effusion in 19 (10.9%) cases, pneumonia in 12 (6.9%) cases, pneumothorax in two (1.1%) cases, atelectasis in four (2.3%) cases, hypoxemia in 11 (6.3%) cases, and respiratory failure in two (1.1%) cases. Some patients developed more than one complication (Fig 1). Three (1.7%) patients required prolonged (>12 h) intubation with mechanical ventilation, and two (1.1%) patients required intensive care unit monitoring and treatment for postoperative pulmonary complications. No patients sustained an intraoperative pleural injury.

**Table 1:** Incidence of postoperative pulmonary complications.

Pulmonary complication	Number of patients	Incidence (%)
Pleural effusion	19	10.9%
Pneumonia	12	6.9%
Pneumothorax	2	1.1%
Atelectasis	4	2.3%
Hypoxemia	11	6.3%
Respiratory failure	2	1.1%
Prolonged intubation with mechanical ventilation	3	1.7%
**Total**	26	14.9%

Compared with patients without postoperative pulmonary complications, patients with postoperative pulmonary complications were significantly older (*P* = 0.001) and had larger preoperative Cobb angles (67.0±2.8° vs. 84.2±9.9°, *P* = 0.002). FVC and FEV_1_ values were lower in patients with than in those without postoperative pulmonary complications (67.1±8.2% vs. 79.6±2.5%, *P* < 0.001 and 64.9±7.9% vs. 75.1±2.5%, *P* = 0.003, respectively). More vertebral levels were fused in patients with than in those without postoperative pulmonary complications (12.8 ± 0.8 vs. 10.8 ± 0.5, *P* < 0.001). Operation times were 432.3±59.9 and 333.4±15.4 min, respectively (*P* = 0.002). Anesthesia times were 544.6±57.8 and 414.8±19.6 min, respectively (*P* < 0.001). Estimated blood loss amounts were 1650.0±423.0 and 976.4±100.7 ml, respectively (*P* = 0.003). Volumes of blood transfusion and intraoperative infusion were 2059.6±435.5 and 951.8±123.8 ml (*P* < 0.001), and 6123.1±908.6 and 4315.3±260.1 ml (*P* < 0.001), respectively. The crystalloid-colloid ratios of intraoperative infusion were 0.9±0.1 and 1.2±0.1, respectively (*P* = 0.002). The proportion of grade 4–6 spinal osteotomies was also higher among patients with than among those without postoperative pulmonary complications (*P* = 0.001). No significant difference in sex, weight, preoperative white blood cell or neutrophil count, intraoperative tidal volume, or intraoperative FiO_2_ was observed (Table 2).

**Table 2:** Baseline clinical and perioperative characteristics of patients with or without pulmonary complications.

Characteristics	Pulmonary Complications		*P*
	Yes (n = 26)	No (n = 148)	
Age (years)	21.1±3.1	15.6±1.0	0.001
Sex			
Males	6 (3%)	40 (23%)	0.674
Females	20 (12%)	108 (62%)	
Weight (kg)	48.9±4.9	43.9±2.2	0.079
Pulmonary function			
FVC (% pred)	67.1±8.2	79.6±2.5	<0.001
FEV_1_ (% pred)	64.9±7.9	75.1±2.5	0.003
Preoperative WBC	5.6±0.4	5.9±0.2	0.266
Preoperative neutrophil	2.8±0.3	3.1±0.2	0.128
Preoperative Cobb angle (degree)	84.2±9.9	67.0±2.8	0.002
Correction of Cobb angle (degree)	52.6±8.2	40.3±2.5	0.002
Spinal osteotomy			
Grade 1–3 osteotomy	12 (7%)	114 (66%)	0.001
Grade 4–6 osteotomy	14 (8%)	34 (19%)	
No. of levels fused	12.8±0.8	10.8±0.5	<0.001
Operation time (min)	432.3±59.9	333.4±15.4	0.002
Anesthesia time (min)	544.6±57.8	414.8±19.6	<0.001
Estimated blood loss (ml)	1650.0±423.0	976.4±100.7	0.003
Volume of blood transfusion (ml)	2059.6±435.5	951.8±123.8	<0.001
Volume of intraoperative infusion (ml)	6123.1±908.6	4315.3±260.1	<0.001
Ratio of crystalloid-colloid	0.9±0.1	1.2±0.1	0.002
Intraoperative tidal volume (ml)	383.8±24.3	362.8±17.0	0.155
Intraoperative FiO_2_ (%)	82.7±9.1	80.8±4.0	0.710

Abbreviation: FVC forced vital capacity, FEV_1_ forced expiratory volume in 1 second, WBC white blood count, No. number, FiO_2_ fraction of inspiration O_2_

**Fig 2:**
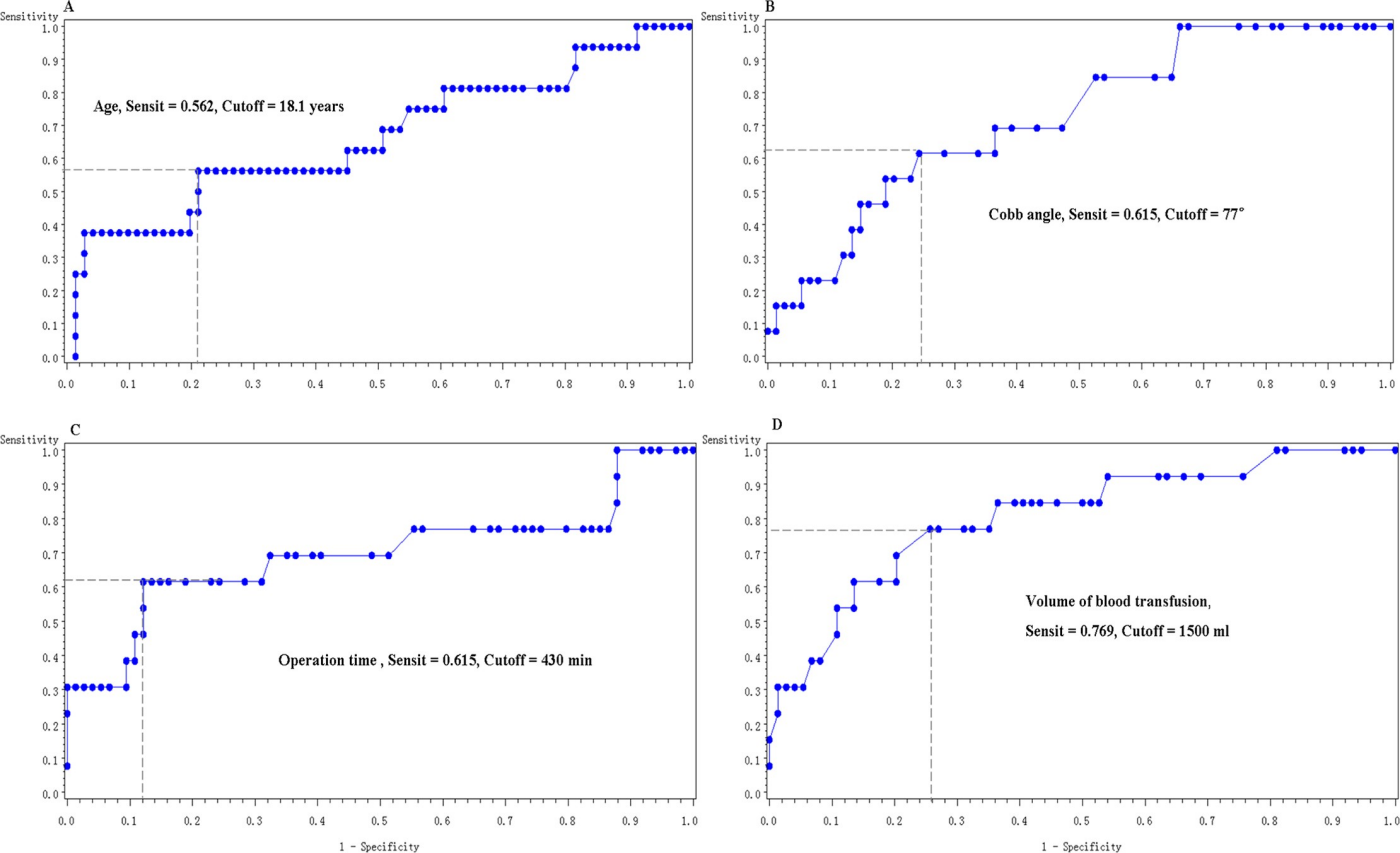
Receiver-operator characteristic curve analyses for the prediction of postoperative pulmonary complication. (A) Age, with a cutoff value of 18.1 years; (B) Cobb angle, with a cutoff value of 77°; (C) operation time, with a cutoff value of 430 min; and (D) blood transfusion, with a cutoff value of 1500 ml.

Age, preoperative Cobb angle, operation time, and volume of blood transfusion were correlated with postoperative pulmonary complications in the logistic regression analysis. Based on the area under the ROC curve in the final prediction model, the optimal cutoff scores for elevated risk were 18.1 years for age, 77° for Cobb angle, 430 min for operation time, and 1500 ml for blood transfusion volume (Fig 2). In other words, most associations with the risk of postoperative pulmonary complications were attributable to an age > 18.1 years (odds ratio [OR] = 2.887, *P* = 0.039), Cobb angle > 77° (OR = 4.338, *P* = 0.011), operation time > 430 min (OR = 3.459, *P* = 0.032), and blood transfusion volume > 1500 ml (OR = 4.212, *P* = 0.015; Table 3). Although no PFT variable was identified as a probable predict factor in the logistic regression analysis, preoperative FVC and FEV_1_ were revealed to be strongly related to age and the Cobb angle. These values were significantly reduced in patients aged >18.1 years (*P* = 0.020 and 0.046, respectively) and those with Cobb angles > 77° (*P* = 0.003 and 0.002, respectively; Table 4).

**Table 3:** Results of multivariate logistic regression analysis regarding the predictive factors for pulmonary complications.

	Estimate	SE	*P* value	Odds ratio	95% CI
Age > 18.1 years	1.060	0.514	0.039	2.887	1.054, 7.904
Cobb angle > 77°	1.467	0.577	0.011	4.338	1.339, 13.451
Operation time > 430 min	1.241	0.578	0.032	3.459	1.114, 10.743
Volume of blood transfusion > 1500 ml	1.438	0.593	0.015	4.212	1.318, 13.459
Constant	-3.328	0.478	<0.001	-	-

Abbreviation: SE standard error, CI confidence interval

**Table 4:** Relationship between pulmonary function, age, and preoperative Cobb angle in patients with congenital scoliosis.

Pulmonary function	Age			Cobb angle		
	≤18.1 years (n = 126)	> 18.1 years (n = 48)	*P*	≤ 77° (n = 122)	> 77° (n = 52)	*P*
FVC (% pred)	80.3±3.8	71.0±8.2	0.020	81.2±3.8	69.7±7.7	0.003
FEV_1_ (% pred)	75.7±3.9	67.9±7.6	0.046	77.1±3.9	65.4±6.8	0.002

Abbreviation: FVC forced vital capacity, FEV_1_ forced expiratory volume in 1 second

## Discussion

CS is one of the most difficult type of early onset scoliosis to manage. This congenital vertebral defect can lead to significant spine deformities, which occasionally present as rigid, severe curvature requiring osteotomy (e.g., SPO, PSO or VCR) to obtain global balance of the spine [9–11]. CS is also often associated with anomalies in other organs, which may be isolated or in association with the VACTERL (vertebral anomalies, anorectal atresia, cardiac anomalies, tracheoesophageal fistula, esophageal atresia, renal and limb anomalies) syndrome [12]. A wide variety of intraspinal abnormalities, including diastematomyelia, intradural lipoma, syringomyelia, Chiari malformation, and tethered cord, is observed in patients with CS [13, 14]. Concurrent abnormities may increase the risk of surgical correction in these patients. Additionally, due to severe thoracic deformity, many patients with CS have pulmonary dysfunction. Previous studies have shown that the incidence of postoperative pulmonary complications is higher in patients with CS and severe restrictive pulmonary dysfunction [4, 15]. In this retrospective, nonrandomized study, the overall incidence rate of postoperative pulmonary complications following posterior spinal instrumentation and fusion surgery for the treatment of CS was 14.9%, indicating that such complications remain a pivotal problem in clinical practice. Our results also indicate that age, preoperative Cobb angle, operation time, and volume of blood transfusion are independent predictive factors for postoperative pulmonary complications in patients with CS.

In the current study, patients older than 18.1 years were 2.887 times as likely to have postoperative pulmonary complications as those younger than 18.1 years. Similarly, Yuan et al. [16] reported that older age (>13 years) was correlated with the requirement for prolonged postoperative mechanical ventilation following scoliosis repair surgery. Patil et al. [17] reported that pediatric patients (age < 18 years) experienced less postoperative complications, including pulmonary complications, than did older patients after surgery to correct idiopathic scoliosis. Kang et al. [6] also demonstrated that patients with neuromuscular scoliosis aged > 16.5 years were more likely to develop postoperative pulmonary complications. These differences may be attributable to the more rigid, severe curvature and sustained restriction of lung growth in adult patients with CS.

According to our logistic regression analysis, Cobb angle > 77° is also an independent risk factor for the development of postoperative pulmonary complications. The incidence of postoperative pulmonary complications in patients with CS and Cobb angles > 77° was 4.338 times that in those with Cobb angle < 77°. Our result is consistent with that of previous study [18], in which Cobb angles > 69° [6] and > 100° were risk factors for postoperative pulmonary complications in patients with neuromuscular scoliosis. A possible reason for this association is that the CS patients with rigid and severe curvature often develop restrictive ventilatory impairment, and pulmonary function damage tends to progressively worsen to combined ventilatory impairment with age. Furthermore, such curvature may increase operation difficulty, operation and anesthesia time, intraoperative blood loss and transfusion, and length of fusion, which also indirectly affect the development of postoperative pulmonary complications. Similarly, the incidence of postoperative pulmonary complications increased with a higher degree of Cobb angle correction postoperatively. The univariate analysis in current study revealed that the degree of Cobb angle correction was significantly greater in patients with than in those without postoperative pulmonary complications (Table 2). However, the objective of this study was to investigate the predictive risk factors of postoperative pulmonary complications following the posterior surgery, and the preoperative Cobb angle was a more appropriate candidate rather than the degree of correction. Thus, because of the high correlation between the two variables, the preoperative Cobb angle was selected as the potential predictive risk factor for the logistic regression analysis rather than the correction of Cobb angle.

Researchers have suggested that a prolonged operative time was associated with increased incidence of delay extubation and pneumonia after multilevel prone spine surgery [19]. Similarly, our study demonstrated a higher incidence of postoperative pulmonary complications among patients with CS undergoing operation lasting > 430 min. Excessively long operation time could lead to prolonged intubation and increased intraoperative blood loss and transfusion, which may be the main reasons for the development of postoperative pulmonary complications. Other operative factors, such as a large transfusion volume, could also be important predictors of pulmonary complications. Massive transfusion is thought to be associated with the development of pulmonary complications because of transfusion-related acute lung injury [20, 21]. In the present study, patients with blood transfusion volumes > 1500 ml were 4.212 times more likely than those with lesser transfusion volumes to have postoperative pulmonary complications.

Theoretical controversy concerning the correlation between preoperative PFT findings and postoperative pulmonary complications persists. Vedantam et al. [3] reported that declining of preoperative pulmonary function was not correlated with the risk of postoperative pulmonary complications in patients with adolescent idiopathic scoliosis. Liang et al. [22] also found that decreased preoperative pulmonary function was not an independent predictor of such complications in patients with scoliosis and moderate to severe pulmonary dysfunction. On the other side, Kang et al. [6] found that a preoperative FVC < 39.5% or FEV_1_ < 40% of the predicted value was a sensitive predictor of the likelihood of postoperative pulmonary complications in patients with neuromuscular scoliosis. Similarly, Lao et al. [15] reported a higher incidence of such complications among patients with severe preoperative pulmonary dysfunction. In our study, decreased preoperative PFT values, including FVC and FEV_1_, were correlated with postoperative pulmonary complications, but were not independent risk factors for the development of such complications, in patients with CS. These findings may reflect interaction effects between preoperative PFT variables and age, Cobb angle, and/or operation time; preoperative FVC and FEV_1_ values decreased with increasing age and preoperative Cobb angle in the present study. Moreover, the relatively small sample did not provide sufficient power to show a statistical correlation between preoperative PFT variables and postoperative pulmonary complications.

It is probably that other factors, such as thoracoplasty, may also affect the incidence of postoperative pulmonary complications in patients with CS. Liang et al. [22] reported that thoracoplasty was the only independent predictor of postoperative pulmonary complications in scoliotic patients with moderate or severe pulmonary dysfunctions. Lao et al [15] found that patients with extremely severe scoliosis who underwent thoracoplasty were more likely than those who didn’t to have postoperative pulmonary complications. However, in consideration of the severe complications after thoracoplasty such as marked decrease in pulmonary function and intraoperative pleural injury [23, 24], the surgeon in this study did not perform thoracoplasty in one-stage with posterior spinal instrumentation and fusion surgery. Thus, thoracoplasty was not a candidate for the predictive risk factors of postoperative pulmonary complications in current study.

Readers should be aware of other limitations of this study. First, this study was retrospective, with a non-randomized and non-blinded design. Second, the results are limited by the relatively small sample and, consequently, limited statistical power. Finally, other limitations may include the lack of consideration of postoperative PFT findings and other potential risk factors, such as surgical experience, intraoperative blood pressure and body temperature, postoperative analgesia, and postoperative distension of the abdomen. A prospective, randomized, multicenter parallel control study is needed to address these issues and confirm our findings.

## Conclusions

Postoperative pulmonary complications are among the main complications following posterior spinal instrumentation and fusion surgery in patients with CS. This study showed that age >18.1 years, Cobb angle > 77°, operation time of > 430 min, and blood transfusion volume > 1500 ml are independent risk factors for the development of these complications following posterior spinal instrumentation and fusion surgery in this patient population.

## Supporting information

S1 File Dataset of this study. (XLS)

S2 File STROBE checklist for this observational study. (DOCX)
