# Supplementary material for: Postoperative pulmonary complications following posterior spinal instrumentation and fusion for congenital scoliosis
Source: PLoS One. 2018 Nov 16;13(11):e0207657. doi: 10.1371/journal.pone.0207657 (PMC6239341; doi:10.1371/journal.pone.0207657)
Supplement: S2 File — (DOCX) [file pone.0207657.s002.docx]

STROBE Statement—checklist of items that should be included in reports of observational studies

|  | Item No. | Recommendation | Page  No. | Relevant text from manuscript |
| --- | --- | --- | --- | --- |
| **Title and abstract** | 1 | (*a*) Indicate the study’s design with a commonly used term in the title or the abstract | 2 | To investigate the incidence of and predictive factors of postoperative pulmonary complications following posterior spinal instrumentation and fusion surgery for the treatment of CS. |
|  |  | (*b*) Provide in the abstract an informative and balanced summary of what was done and what was found | 2-3 | We retrospectively reviewed the records of 174 patients with CS (128 females and 46 males, mean age 16.4 years) treated with posterior spinal instrumentation and fusion surgery between January 2012 and April 2017. We extracted demographic, medical history, and clinical data, and investigated the major predictive factors for postoperative pulmonary complications by logistic regression and receiver-operating characteristic curves analyses.  Postoperative pulmonary complications are among the main complications following posterior spinal instrumentation and fusion surgery in patients with CS. Such patients aged >18.1 years, with Cobb angles > 77°, operation times > 430 min, and/or blood transfusion volume of > 1500 ml may be more likely to develop postoperative pulmonary complications. |
| Introduction | | | |  |
| Background/rationale | 2 | Explain the scientific background and rationale for the investigation being reported | 4 | Little has been reported about the postoperative pulmonary complication events during initial hospitalization in patients with CS. |
| Objectives | 3 | State specific objectives, including any prespecified hypotheses | 4 | This retrospective study was undertaken to investigate the prevalence of and predictive factors related to postoperative pulmonary complications following posterior spinal instrumentation and fusion surgery for the treatment of CS. |
| Methods | | | |  |
| Study design | 4 | Present key elements of study design early in the paper | 4-7 | Patients  Clinical and radiological assessment  Surgical techniques  Pulmonary complications |
| Setting | 5 | Describe the setting, locations, and relevant dates, including periods of recruitment, exposure, follow-up, and data collection | 4 | We reviewed the hospital and clinical records of 174 patients with CS (46 males, 128 females; mean age 16.4 years, range 4.8-44.6 years) who underwent posterior spinal instrumentation and fusion surgery between January 2012 and April 2017. |
| Participants | 6 | (*a*) *Cohort study*—Give the eligibility criteria, and the sources and methods of selection of participants. Describe methods of follow-up  *Case-control study*—Give the eligibility criteria, and the sources and methods of case ascertainment and control selection. Give the rationale for the choice of cases and controls  *Cross-sectional study*—Give the eligibility criteria, and the sources and methods of selection of participants | 4 | We reviewed the hospital and clinical records of 174 patients with CS (46 males, 128 females; mean age 16.4 years, range 4.8-44.6 years) who underwent posterior spinal instrumentation and fusion surgery between January 2012 and April 2017. |
|  |  | (*b*) *Cohort study*—For matched studies, give matching criteria and number of exposed and unexposed  *Case-control study*—For matched studies, give matching criteria and the number of controls per case |  |  |
| Variables | 7 | Clearly define all outcomes, exposures, predictors, potential confounders, and effect modifiers. Give diagnostic criteria, if applicable | 5 | Before surgery, patients’ preoperative conditions were assessed by a series of examinations including a complete blood cell count, measurement of the erythrocyte sedimentation rate and C-reactive protein level, pulmonary function testing (PFT), and abdominal ultrasound and echocardiography. PFT measured five parameters: forced vital capacity (FVC), the FVC ratio, forced expiratory volume (FEV), and FEV at the end of the first second (FEV1) and its ratio.  Preoperative radiographs of the patients, including chest X-ray, standing anteroposterior and lateral radiographs of the entire spine, whole-spine magnetic resonance (MR) images, three-dimensional computed tomography (CT) reconstructions, and supine right and left bending radiographs, were also examined. |
| Data sources/ measurement | 8* | For each variable of interest, give sources of data and details of methods of assessment (measurement). Describe comparability of assessment methods if there is more than one group | 5-7, 20 | Cobb angles and coronal and sagittal alignments were measured on long cassette films using C7 as the plumb line reference. Postoperative radiographic data included the degree of spinal curvature correction immediately postoperatively and at the final follow-up.  Postoperative pulmonary complications were defined as pulmonary abnormalities occurring in the postoperative period, including atelectasis, pleural effusion, pneumothorax, pneumonia, hypoxemia (oxygen saturation (SO2) < 90% over 8 h), respiratory failure, and increased requirement for postoperative mechanical ventilation. The presence of any perioperative cardiopulmonary symptoms or signs (dyspnea, breathlessness on exertion, crackles, or rhonchi) were recorded. Chest radiographs obtained within 3 days postoperatively were examined to note the presence of pulmonary complications. Postoperative chest radiographs and thoracic ultrasound images were obtained in patients with abnormal cardiopulmonary symptoms and signs or suspicious chest auscultation findings, when necessary.  S1 File. Dataset of this study. (XLSX) |
| Bias | 9 | Describe any efforts to address potential sources of bias | 5-6 | The same surgeon (H Tao) performed all surgeries. After thoracic vertebral osteotomy, an intraoperative lung recruitment maneuver was routinely conducted to confirm that no intraoperative pleural injury was detected.All patients were closely monitored intraoperatively according to transcranial electric motor-evoked potential and somatosensory-evoked potential. After surgery, all patients were engaged in a supervised physical therapy program and given a home exercise protocol. |
| Study size | 10 | Explain how the study size was arrived at | N/A |  |

Continued on next page

| Quantitative variables | 11 | Explain how quantitative variables were handled in the analyses. If applicable, describe which groupings were chosen and why | 7 | Univariate analysis with the t test was performed to examine potential risk factors among continuous variables, such as age, weight, PFT variables, blood cell count variables, Cobb angle, fusion levels, operation time, duration of anesthesia, estimated blood loss, volume of blood transfusion and intraoperative infusion, crystalloid-colloid ratio, intraoperative fraction of oxygen inspiration (FiO2), and intraoperative tidal volume. Univariate analysis by the chi-square test and Fisher’s exact test was performed to examine potential risk factors among categorical variables, such as sex, grade of osteotomy, and preoperative chest X-ray findings. A multiple logistic regression model was used to identify significant predictors of postoperative pulmonary complications. |
| --- | --- | --- | --- | --- |
| Statistical methods | 12 | (*a*) Describe all statistical methods, including those used to control for confounding | 7-8 | We generated a receiver-operating characteristic (ROC) curve using predicted probability values from the logistic regression model. The ROC curve was used to evaluate the optimal cutoff value, which was calculated based on the maximal sum of sensitivity and specificity with the bootstrap normal approximation method. |
|  |  | (*b*) Describe any methods used to examine subgroups and interactions | N/A |  |
|  |  | (*c*) Explain how missing data were addressed | 8 | No patient was lost to follow-up. The mean follow-up duration was 27 months (range 6-69 months). |
|  |  | (*d*) *Cohort study*—If applicable, explain how loss to follow-up was addressed  *Case-control study*—If applicable, explain how matching of cases and controls was addressed  *Cross-sectional study*—If applicable, describe analytical methods taking account of sampling strategy | 7-8 | Univariate analysis by the chi-square test and Fisher’s exact test was performed to examine potential risk factors among categorical variables, such as sex, grade of osteotomy, and preoperative chest X-ray findings. A multiple logistic regression model was used to identify significant predictors of postoperative pulmonary complications. We generated a receiver-operating characteristic (ROC) curve using predicted probability values from the logistic regression model. The ROC curve was used to evaluate the optimal cutoff value, which was calculated based on the maximal sum of sensitivity and specificity with the bootstrap normal approximation method. |
|  |  | (*e*) Describe any sensitivity analyses | N/A |  |
| Results | | | | |
| Participants | 13* | (a) Report numbers of individuals at each stage of study—eg numbers potentially eligible, examined for eligibility, confirmed eligible, included in the study, completing follow-up, and analysed | 8 | No patient was lost to follow-up. The mean follow-up duration was 27 months (range 6-69 months). The mean operation time was 348.2 min (range 145-660 min), with an average estimated blood loss of 1077.0 ml (range 300-3500 ml). The average number of levels fused during operation was 11.1 (range 5-15). The average preoperative coronal Cobb angle was 69.6° (range 45-144°), which was corrected to 29.1° (range 18-52°) immediately postoperatively, with a correction rate of 55.1%. No obvious loss of correction was observed at final follow-up examinations. |
|  |  | (b) Give reasons for non-participation at each stage | 8 | No patients were lost to follow-up. |
|  |  | (c) Consider use of a flow diagram | N/A |  |
| Descriptive data | 14* | (a) Give characteristics of study participants (eg demographic, clinical, social) and information on exposures and potential confounders | 8-9 | The incidence of postoperative pulmonary complications is shown in Table 1. Twenty-six (14.9%) of the 174 patients, developed postoperative pulmonary complications, consisting of pleural effusion in 19 (10.9%) cases, pneumonia in 12 (6.9%) cases, pneumothorax in two (1.1%) cases, atelectasis in four (2.3%) cases, hypoxemia in 11 (6.3%) cases, and respiratory failure in two (1.1%) cases. Some patients developed more than one complication (Fig 1). Three (1.7%) patients required prolonged (>12 h) intubation with mechanical ventilation, and two (1.1%) patients required intensive care unit monitoring and treatment for postoperative pulmonary complications. No patients sustained an intraoperative pleural injury. |
|  |  | (b) Indicate number of participants with missing data for each variable of interest | 8 | No patients were lost to follow-up. |
|  |  | (c) *Cohort study*—Summarise follow-up time (eg, average and total amount) | 8 | The mean follow-up was 27 months (range 6-69 months). |
| Outcome data | 15* | Cohort study—Report numbers of outcome events or summary measures over time | 8-9 | Twenty-six (14.9%) of the 174 patients, developed postoperative pulmonary complications, consisting of pleural effusion in 19 (10.9%) cases, pneumonia in 12 (6.9%) cases, pneumothorax in two (1.1%) cases, atelectasis in four (2.3%) cases, hypoxemia in 11 (6.3%) cases, and respiratory failure in two (1.1%) cases. Some patients developed more than one complication (Fig 1). Three (1.7%) patients required prolonged (>12 h) intubation with mechanical ventilation, and two (1.1%) patients required intensive care unit monitoring and treatment for postoperative pulmonary complications. |
|  |  | *Case-control study—*Report numbers in each exposure category, or summary measures of exposure |  |  |
|  |  | *Cross-sectional study—*Report numbers of outcome events or summary measures |  |  |
| Main results | 16 | (*a*) Give unadjusted estimates and, if applicable, confounder-adjusted estimates and their precision (eg, 95% confidence interval). Make clear which confounders were adjusted for and why they were included | N/A |  |
|  |  | (*b*) Report category boundaries when continuous variables were categorized | N/A |  |
|  |  | (c) If relevant, consider translating estimates of relative risk into absolute risk for a meaningful time period | 13 | Age, preoperative Cobb angle, operation time, and volume of blood transfusion were correlated with postoperative pulmonary complications in the logistic regression analysis. Based on the area under the ROC curve in the final prediction model, the optimal cutoff scores for elevated risk were 18.1 years for age, 77° for Cobb angle, 430 min for operation time, and 1500 ml for blood transfusion volume (Fig 2). In other words, most associations with the risk of postoperative pulmonary complications were attributable to an age > 18.1 years (odds ratio [OR] = 2.887, P = 0.039), Cobb angle > 77° (OR = 4.338, P = 0.011), operation time > 430 min (OR = 3.459, P = 0.032), and blood transfusion volume > 1500 ml (OR = 4.212, P = 0.015; Table 3). |

Continued on next page

| Other analyses | 17 | Report other analyses done—eg analyses of subgroups and interactions, and sensitivity analyses | N/A |  |
| --- | --- | --- | --- | --- |
| Discussion | | | | |
| Key results | 18 | Summarise key results with reference to study objectives | 16-18 | In the current study, patients older than 18.1 years were 2.887 times as likely to have postoperative pulmonary complications as those younger than 18.1 years.  According to our logistic regression analysis, Cobb angle > 77° is also an independent risk factor for the development of postoperative pulmonary complications.  Similarly, our study demonstrated a higher incidence of postoperative pulmonary complications among patients with CS undergoing operation lasting > 430 min.  In the present study, patients with blood transfusion volumes > 1500 ml were 4.212 times more likely than those with lesser transfusion volumes to have postoperative pulmonary complications. |
| Limitations | 19 | Discuss limitations of the study, taking into account sources of potential bias or imprecision. Discuss both direction and magnitude of any potential bias | 19 | Readers should be aware of other limitations of this study. First, this study was retrospective, with a non-randomized and non-blinded design. Second, the results are limited by the relatively small sample and, consequently, limited statistical power. Finally, other limitations may include the lack of consideration of postoperative PFT findings and other potential risk factors, such as surgical experience, intraoperative blood pressure and body temperature, postoperative analgesia, and postoperative distension of the abdomen. A prospective, randomized, multicentre parallel control study is needed to address these issues and confirm our findings. |
| Interpretation | 20 | Give a cautious overall interpretation of results considering objectives, limitations, multiplicity of analyses, results from similar studies, and other relevant evidence | 20 | Postoperative pulmonary complications are among the main complications following posterior spinal instrumentation and fusion surgery in patients with CS. This study showed that age >18.1 years, Cobb angle > 77°, operation time of > 430 min, and blood transfusion volume > 1500 ml are independent risk factors for the development of these complications following posterior spinal instrumentation and fusion surgery in this patient population. |
| Generalisability | 21 | Discuss the generalisability (external validity) of the study results | 20 | This study showed that age >18.1 years, Cobb angle > 77°, operation time of > 430 min, and blood transfusion volume > 1500 ml are independent risk factors for the development of these complications following posterior spinal instrumentation and fusion surgery in this patient population. |
| Other information | |  | | |
| Funding | 22 | Give the source of funding and the role of the funders for the present study and, if applicable, for the original study on which the present article is based | 15 | N/A |

*Give information separately for cases and controls in case-control studies and, if applicable, for exposed and unexposed groups in cohort and cross-sectional studies.

Note: An Explanation and Elaboration article discusses each checklist item and gives methodological background and published examples of transparent reporting. The STROBE checklist is best used in conjunction with this article (freely available on the Web sites of PLoS Medicine at http://www.plosmedicine.org/, Annals of Internal Medicine at http://www.annals.org/, and Epidemiology at http://www.epidem.com/). Information on the STROBE Initiative is available at www.strobe-statement.org.
